# Personal Data Stores (PDS): A Review

**DOI:** 10.3390/s23031477

**Published:** 2023-01-28

**Authors:** Khalid U. Fallatah, Mahmoud Barhamgi, Charith Perera

**Affiliations:** 1 Faculty of Applied Sciences, Shaqra University, Riyadh 15526, Saudi Arabia; 2 Department of Computer Science and Engineering, Qatar University, Doha P.O. Box 2713, Qatar; 3 School of Computer Science and Informatics, Cardiff University, Cardiff CF24 4AG, UK

**Keywords:** internet of things, personal data store, data vaults, personal data management, personal informatics

## Abstract

Internet services have collected our personal data since their inception. In the beginning, the personal data collection was uncoordinated and was limited to a few selected data types such as names, ages, birthdays, etc. Due to the widespread use of social media, more and more personal data has been collected by different online services. We increasingly see that Internet of Things (IoT) devices are also being adopted by consumers, making it possible for companies to capture personal data (including very sensitive data) with much less effort and autonomously at a very low cost. Current systems architectures aim to collect, store, and process our personal data in the cloud with very limited control when it comes to giving back to citizens. However, Personal Data Stores (PDS) have been proposed as an alternative architecture where personal data will be stored within households, giving us complete control (self-sovereignty) over our data. This paper surveys the current literature on Personal Data Stores (PDS) that enable individuals to collect, control, store, and manage their data. In particular, we provide a comprehensive review of related concepts and the expected benefits of PDS platforms. Further, we compare and analyse existing PDS platforms in terms of their capabilities and core components. Subsequently, we summarise the major challenges and issues facing PDS platforms’ development and widespread adoption.

## 1. Introduction

The technological advancement in the daily lives of individuals has increased the creation, exchange, and use of personal data to levels we have never seen before. Social media platforms alone are responsible for creating a big part of this data since more than 4.2 billion people are daily using these platforms [1]. Other online web services (e.g., search engines, emails, digital file storage, etc.) also generate massive amounts of data. In addition to that, with the pervasiveness of IoT technologies, billions of smart objects (e.g., sensors, home appliances, cameras, etc.) are designed to generate and collect a wealth of personal data [2].

However, despite the tremendous benefits of using these technologies, there are growing concerns and challenges regarding the control and ownership of personal data [3]. While control of personal data refers to the ability to collect, organise, protect, and store the data, ownership refers to having the right and ability to create economic and social value [4]. In the current centralised Internet infrastructure, individuals have little or no control over the storage and usage of their data [5]. Furthermore, with this centralised structure, personal data are vulnerable to data security and privacy issues (e.g., data breaches by Facebook) and unlawful usage of the data [6]. Besides, individuals will not be able to use their data as a valuable asset to create profit.

With the emergence of the Personal Data Store (PDS) and the introduction of the General Data Protection Regulation (GDPR), the focus has shifted from a service provider-centric model to a user-centric model as a potential solution for the challenges mentioned above. In the user-centric model, individuals have full control and ownership over their data. This means they have the right to collect, self-manage, and exchange their data. PDS platforms are designed to achieve this by allowing individuals to aggregate scattered data from different online systems (e.g., social media, banks, hospitals, airlines, etc.) and provide the tools needed to manage and share their data [7]. In addition, these PDS platforms allow individuals to create value for their data by providing tools for data trading based on their privacy preferences and permissions [4].

Currently, most of the research related to the PDS model has focused on the development of PDS platforms that enforce user privacy preferences [8,9], and provide capabilities to store and share personal data [10,11,12]. However, no previous research study has offered a comprehensive review of current PDS platforms within the academic literature. Thus, we aim to fill this gap in the literature by posing the following question (RQ): What is the current research and development status on personal data stores regarding architectures, capabilities, and challenges?

In order to answer this research question, we review the related literature and provide the most recent research development of PDS platforms. Our review covers the theoretical advantages and disadvantages of PDS technology as an alternative solution for a user-centric model for individuals to regain control over personal data. Further, we discuss the meaning, types, and value of personal data that exist in the online environment. Therefore, our contributions can be summarised as follows:

- We review the recent research related to the developments of Personal Data Stores, covering their benefits, capabilities, and architectural design;
- We elaborate, compare, and analyse the capabilities and the architectural design of existing PDS platforms;
- We discuss the open problems and challenges that face the development and adoption of PDS platforms and outline some important future research directions.

The rest of this paper is organised as follows: The paper begins with a brief discussion on the background of personal data in Section 2. It then explores the direct and indirect value of personal data. Then, Section 3 describes the research methods we used to review and search the current literature. Next, Section 4 explains the fundamental idea of personal data store, the expected advantages and disadvantages of the PDS model, and the key enabling technologies for PDS platforms and existing PDS Platforms. Section 5 profoundly investigates the capabilities of PDS platforms, along with their architectures and components in Section 6. We then provide brief discussions in Section 7. Next, Section 8 presents different types of challenges facing the development of PDS platforms. Finally, Section 9 discusses a few lessons learned from the literature, and the Conclusion follows in Section 12.

## 2. Research Background on Personal Data

### 2.1. An Overview of Personal Data, Dimensions, and Its Value

Personal data refers to a vital aspect of our digital world. Some may refer to personal data as photos, emails, and digital footprints. However, personal data involves more than that. According to Ref. [13] personal data is defined as "any information relating to an identified or identifiable natural person (‘data subject’)"; an identifiable person is the one who can be identified, directly or indirectly, in particular by reference to an identification number or to one or more factors specific to his physical, physiological, mental, economic, cultural or social identity. Van [14] uses a unique approach to define personal data as any the information over which a person has some interest or control to negotiate their environment or order their lives. Based on this definition, personal data refers to various things in different disciplines and communities.

In literature, however, personal data can be categorised into three types based on its origin [15]. One is the volunteered data, which is provided or created by individuals (e.g., photos, emails, tweets, and online transaction data and others). The second type is the observed data, such as internet browsing preferences, surveillance video, location, call detail records, and others. Finally, there is inferred data where computational analysis is used, such as credit scores, consumer profiles, predictive traffic flows, and targeted advertisements [13].

**Figure 1:**
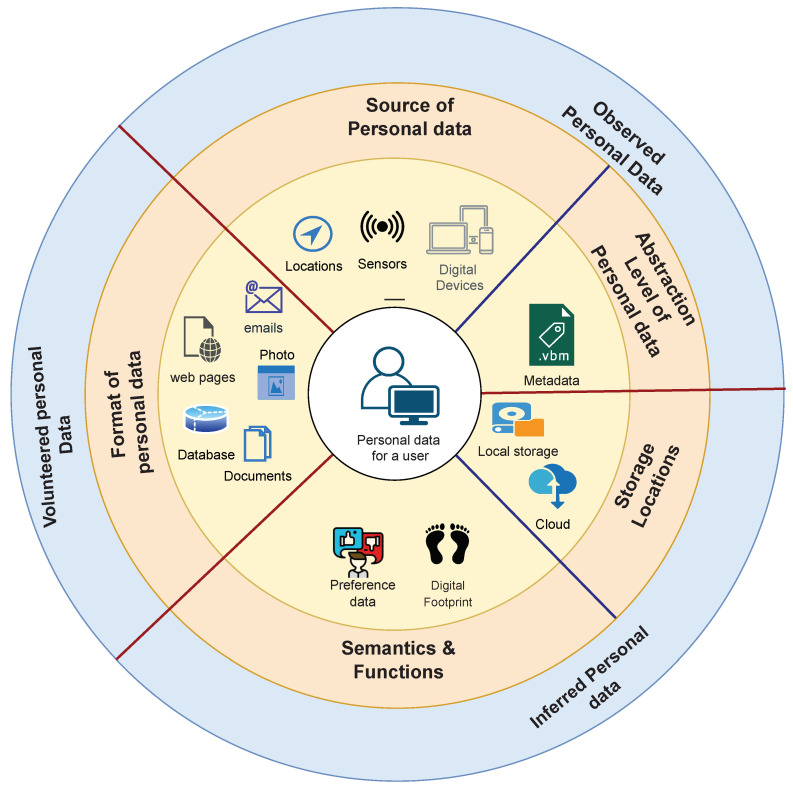
Two levels of classification of Personal Data (PD) [15,16]. Level one involves volunteered data (intentionally created by a user), Observed PD (created automatically about a user), and Inferred PD (generated after computational analysis). Level two involves format, source, abstraction level, storage location and semantics, and functions of PD.

According to Ref. [16], personal data can also be classified into multiple dimensions. The first dimension is the format, which includes documents, multimedia, web pages, email, and database. The second dimension is named the source, which refers to where personal data is generated, including but not limited to personal devices, social media, and sensors. The third dimension is the abstraction level of personal data, including metadata and instance data. The fourth dimension is the semantics and functions, which are about data preference, web footprints, and others. Finally, the last dimension is related to the storage location, including local, distributed, and centralised cloud storage. As shown in Figure 1, both classifications can be combined to provide a comprehensive picture of our data.

### 2.2. The Value of Personal Data from the User’s Perspective

Individuals will gain direct and indirect value with a practical ability to control, protect, and share personal data. Eventually, online services that provide no tools or methods to control personal data might be neglected and abandoned. Therefore, some research works provide initial ideas about how people will trade and sell their data. For example, Ref. [17] propose a subscription service that allows individuals to directly and explicitly sell their data to interested buyers. The proposed subscription involves different data packages. The price of these packages is determined by the data sensitivity level (the more sensitive, the more expensive). Another mechanism is proposed to guide individuals to trade data without allowing agents to access private personal data. Regarding data trading, Ref. [18] has introduced an iterative auction mechanism used by various agents (data owners, collectors, and users) to coordinate the data trading among those agents. In the context of the IoT ecosystem, Ref. [19] proposed sensing as a service model. This business model enables individuals to exchange their data (e.g., trade) with data consumers (e.g., companies and governments).

The value of personal data can be realised in the user-centric model. This model aims to enable individuals to control the process of personal data collection, management, use, and sharing with others [20]. Chessa and Loiseau [21] have introduced a cooperative personal data store (CPDS) model for managing social network data. In this model, the CPDS works as an intermediary between users and online services that collects personal data and relationships of users who opt-in, selects an efficient data disclosure profile, and appropriately rewards users. This research aims to quantify the value of personal data contributed by each user to establish a fair and efficient reward mechanism. The user-centric model also provides a context where rules and policies are deployed to enforce the fundamental principles that individuals care about, such as trust and transparency [20,22].

The value of personal data can be viewed in the following:

- Personal data represents the Internet footprints of individuals. The size of such data gradually grows as they use various online services and mobile devices daily. Service providers automatically generate, track, and record these types of data. Very sophisticated tools will then be used for aggregating and analysing the footprints for a deeper understanding of users’ behaviours.
- Personal data is the e-history of individuals. Nowadays, people intensively use social media and other online services from an early age [23]. They also depend on many of these services for social interactions. With a practical ability to control personal data, individuals will become able to view and summarise crucial parts of their history.
- Using personal data can be used to offer and provide personalised online services and advertisements ideally.

## 3. Research Methodology

### 3.1. Search Process and Paper Selection

The aim of surveying all publications related to the development of PDS implies the need to go through a careful and comprehensive search process. The process involves several steps, which will be explained below:

To begin with, we include all papers that discuss and address any aspects of PDS, such as benefits, functions, architecture, challenges, etc. We also use only papers written in English and published as conference papers, journal papers, theses, technical reports, or books. So far, we have performed two types of searches on related publications published from 2000:

- Using online library search including major search engines: ACM Digital Library, IEEE Xplore Digital Library, Elsevier ScienceDirect, and Google scholar. As shown in Table 1, we list all the used search terms and their combinations.
- Reference list search for identifying papers missed in the previous step (backward and forward search).

We carefully read each publication’s title and abstract (and relevant sections when necessary). In case of insufficient information in the title and abstracts to make a decision, we further reviewed the full text of the paper. This step is critical to exclude irrelevant papers that did not meet the aim of this report. Then, we manually filter out unrelated publications. Later, key authors might be contacted via email to check whether we have covered all important references and the accuracy of information regarding our descriptions of their works.

### 3.2. Research Analysis

To classify topics related to PDS in this survey, we first analysed all the collected papers. Based on this analysis, we found that research aspects can be categorised into two top-level categories: theoretical and technical aspects. Each category was then divided into sub-categories based on the correlation to the top-level categories. Table 2 presents a detailed taxonomy of research related to personal data stores. The collected papers were then manually classified and assigned to each sub-category.

As we mentioned above, the research landscape of PDS can be viewed in two ways: theoretical and technical aspects. The former focuses on what has been directly published in the literature regarding the adoption and development of the data store model. The latter view the technical aspects of PDS platforms and some variables that might be used to solve the technical issues facing the development of PDS platforms.

## 4. Evolution of Personal Data Store

The idea of the personal data store goes back to the early 2000s when Ref. [48] introduced the concept of a personal digital store. The initial idea of this concept was to store and capture digital materials (e.g., books, photos, and other digital documents). This idea was developed for MyLifeBits as a platform to store scanned paper files and record, store, and access a personal lifetime archive [49]. Personal web observatories are another concept based on the idea of PDS [50]. A personal web observatory is a technical platform that, first and foremost, enables individuals to consolidate and archive their data that is dispersed among multiple sources. Later, the concept of Personal Information Management (PIM) [33] and Personal dataspace management [51] was introduced to specifically focus on the process of managing personal digital information such as emails, images, HTML, XML, audio, video, and so on. However, these concepts merely focus on how an individual manages his or her data and ignore the capability of sharing or even trading their data with other entities (data consumers) to gain returned values [52].

### 4.1. Privacy as a Driver for PDS to Flourish

A personal Data Store can be described as a model, framework, architecture, or ecosystem designed to give individuals ultimate control over their personal data. A person could collect, store, manage, and share his data according to his rules [5]. This definition has focused only on the fundamental processes that PDSs should have. However, other researchers further provide more details to describe PDS platforms. According to [53], a PDS is defined as “a set of capabilities built into a software platform or service that allows an individual to manage and maintain his or her digital information, artefacts and assets, longitudinally and self-sufficiently, so it may be used practically when and where it can form the individual’s benefit as perceived by the individual, and shared with others directly, without relying on external third parties”.

Furthermore, recent research initiatives have proposed better forms of PDS that empower individuals to own, control, manage, and share their personal data. The PDS model is fundamentally designed to give individuals the ability to have complete control over their data [54]. As a result, different terms have been introduced in the literature, such as Personal Data Stores (PDSs), Databox, Data Hub, Personal Information Hub, Personal Data Vaults, Personal Container, Smart Hubs, and Home Hubs.

### 4.2. Data Sovereignty as a Legal Requirement

Data sovereignty is another relevant concept to the PDS model, which is defined as the capability for individuals to have full control and determine restrictions and rules about the usage of their data (e.g., access control authorisation and usage duration) before sharing it with data consumers [55,56]. Additionally, all potential data consumers need to be transparent with the data owner. Recently, the Industrial Data Space (IDS) standard initiative proposed a reference architecture model [57]. Based on this model, data sovereignty has been considered a prerequisite for the personal data ecosystem where individuals have the ability to exploit their data as an asset for creating business opportunities for data producers and data consumers.

### 4.3. The Anticipated Advantages of PDS Model

One of the PDS model’s most prominent benefits is user empowerment. Empowering users means the ability for individuals to collect, analyse, manage, and share it with others. This also leads them to regain complete control over data processing. As a result, individuals need to give their consent for data processing and be better informed about it (e.g., potential risks, real-time logs, audits, monitoring, and visualisations). Empowerment would allow individuals to better understand how their data is being processed and feel empowered by using controlling tools provided by PDS platforms. It could also increase the trust of individuals to be more engaged in online transitions.

The second benefit would be the ability for individuals to increase the level of security by determining what, who, and when personal data can be accessed and shared [5]. Besides, regular leakages and privacy issues of even big and popular cloud-based data silos can be minimised by using the PDS model. This would be very useful to enable a decentralised platform that encourages third-party and app developers to embrace more privacy-friendly approaches [53]. Furthermore, a decentralised platform would enable new applications that combine data from many silos to draw inferences unavailable in the existing marketplace [38]. According to the literature, this model could solve and lessen many of today’s issues and concerns related to privacy and data protection.

The PDS model could also be a viable solution for organisations and app developers to access a wide range of personal data (e.g., medical data, bank statements, shopping history, or fitness activities) that would be difficult, or illegal to be collected using current means. In addition to that, once the model is appropriately deployed, online service providers could easily transfer data (with data subject permission). This would then allow organisations (data consumers) to have clean, rich, and safe data. This is a dream come true for third parties, including big organisations and app developers, to perform computations and analytics with clean and rich data. Organisations could also reduce the burdens associated with acquiring and managing individuals’ data.

Another promising benefit of PDS architecture is that individuals will eventually gain the capability to make profits by monetising their personal data. PDS platforms, many of which are under development, have proposed various business models to achieve this feature. For instance, some of these platforms ask data consumers (e.g., app developers) to pay per data transaction, and the type of personal data determines the price. This means an app developer could access an individual’s data once consent is approved. Other platforms (e.g., PDS Mydex) require app developers to pay registration fees to be part of the PDS’ ecosystem and access individuals’ personal data. Alternatively, payments could also be required when app developers need to transfer and/or collective computations [24]. In return, individuals will earn small cash, discounts, or other rewards when they share their personal data.

Finally, PDS architecture is expected to provide the tools that enable individuals to analyse their personal data and gain insights about themselves. The ability to self-quantify, self-knowledge, or self-reflect has become possible due to personal informatics tools and the improved sensor technology [58]. At first, research in this area mainly focused on the utility of personal informatics. Other researchers went beyond that to suggest concentrating on the role and experience of living with data (‘lived’ informatics) [29,30,59]. To define personal informatics (PI), Li and Forlizzi [60] conducted surveys and interviews with people who collect and reflect on personal information. They define PI as systems that assist people in collecting relevant information intending to reflect and gain knowledge about themselves. A stage-based model was derived, in which five stages were discussed (preparation, collection, integration, reflection, and action). Some research works have developed methods that assist individuals in making sense of live data derived from smart home sensors [61,62] and reflect on their personal data and gain insights. Choe [63] built a web-based application called Visualised Self that helps users visualise and explore data. Feustel [64] examined how individuals make sense of their own data when it is presented alongside others’ aggregated data. This research work investigated how people could integrate the data of others to make sense of their own data and how they identify insights and form goals without pre-existing social ties.

### 4.4. The Disadvantages of the PDS Model

As discussed previously, the PDS model provides multiple sensible benefits for individuals regarding data protection, data sovereignty, and privacy. However, this model introduces several drawbacks that may prevent individuals from realising these benefits. The main drawback is that a potential increase of responsibility may be laid on individuals to manage and control their data, particularly for those who are not technically savvy. This also includes the burden to give and manage access and consent for data consumers, which may lead to privacy risks and unintended consequences [8]. Another important issue is data availability and accessibility, especially for local-based PDS platforms. Individuals need to securely access their personal data from anywhere and anytime. In addition, current PDS platforms are still in the early development stages and do not follow technical standards. Each platform has different security and privacy policies, terms of service, functionalities, used technologies, and systems. Thus, this may require individuals to spend a lot of time and effort before they realise the value of using PDS platforms.

### 4.5. Smart Home Platforms as a PDS

The smart home platform (SHP) is a digital home system that enables a homeowner to control, optimise, and monitor some home functions such as thermostats, lighting, air conditions, security systems, and others. These functions can be managed using software called Platforms, which act as the backbone of this digital ecosystem. A typical smart home platform is built to integrate a heterogeneous set of physical devices from various brands, such as Nest thermostats, security cameras, or smart lighting bulbs. With all these devices in place, individuals manage each device using a mobile application. This application will then allow a user to create, edit, or even delete different types of routines and automatic rules such as trigger-action routines (e.g., warn me if there is activity at my living room, turn the air condition on when I am heading home) and scheduled routines (e.g., open the curtain at my bedroom with sunrise and everyday switch all lights off at 8:00 p.m.). However, using SHP allows homeowners to have central control over multiple devices and a unified interface for accessing sensor data. Another essential feature of the smart home platform is the increase of interoperability and connectivity between smart home devices by using various proposed solutions such as a unified control platform or an open IoT platform [65,66]. As a result, users could connect smart devices from a wide range of manufacturers easily. What makes smart home platforms more fascinating is their ability to collect data related to motion, temperature, lighting control, and the state of smart devices [67,68]. This data can be handy for individuals to self-reflect and self-monitor.

Nevertheless, collecting meaningful data from smart home platforms would be challenging because they have different data storage methods [67]. In addition, smart home platforms do not provide technological solutions for individuals to store and analysis personal data. In contrast, PDS platforms are designed to collect, store, and analyse personal data from different sources. Therefore, it would be realistic and motivating to convert a smart home platform into a PDS platform. By doing so, individuals could take advantage of both platforms and can store and collect a large amount of data related to their smart home devices. Then, they would be able to use the collected data for personal analytics and data trading.

**Figure 2:**
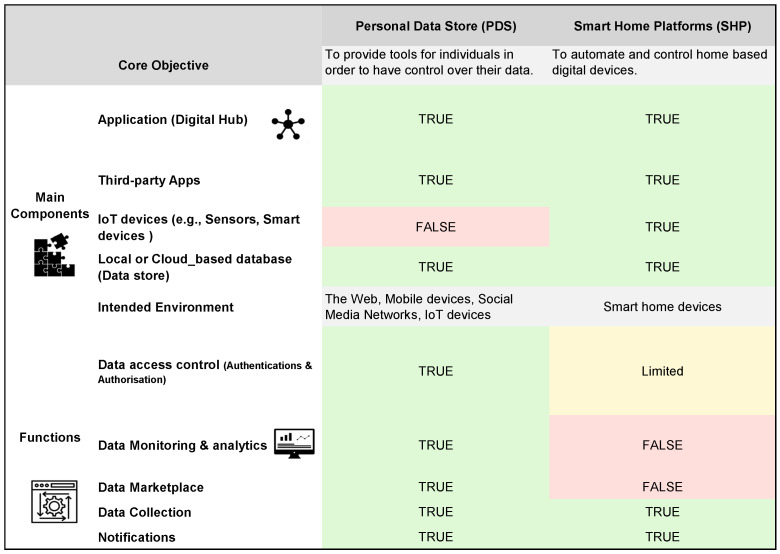
Similarities and differences between PDS platforms and Smart Home Platforms.

Regarding the main components and functions, SHP platforms share some similarities to PDS platforms, which can be seen in Figure 2. According to Kafle [69], the general architecture of smart home platforms consists of apps, devices (e.g., sensors, lighting bulbs, smart speakers, etc.), and centralised data stores where added sensors, rules, routines, and state variables of the entire smart home are stored. These components typically communicate locally over Wi-Fi networks or over the Internet. However, unlike PDS platforms, which is focused on providing the best control over personal data, smart home platforms are essentially designed to automate various aspects of physical devices ranging from small devices with little computing power to large appliances such as refrigerators.

### 4.6. Using PDS Platforms for Enabling Personal Data Marketplace

With the new EU General Data Protection Regulation (GDPR), individuals have become more than ever able to collect, transfer, store, and even trade their personal data. Under these new regulations, individuals have the right to transfer their data collected by firms and other service providers. However, without the use of PDS platforms, it would be difficult for individuals and data consumers to exchange data and create mutual value since there are technical challenges that both sides would face. Therefore, PDS platforms are designed and engineered to overcome these challenges by creating decentralised data marketplaces that enable all parties to share and trade personal data in several ways.

The first way is to ensure the supply of personal data by allowing individuals to gain and retrieve their data from big firms or service providers (e.g., Digi.me). This is because, currently, firms or service providers collect and own personal data. Second, PDS platforms provide tools that individuals can use to manage and control their data. This includes their ability to short, search, and transfer personal data analysis in order to transform personal data into meaningful information. Third, PDS platforms enable individuals to specify and reconfigure their security, privacy, and sharing preferences regarding data sharing and access control. Finally, PDS platforms can be seen as a potential enablers for the data-sharing marketplace because they will ultimately need to provide methods and a virtual environment where data consumers can request and negotiate access to individuals’ personal data. In contrast, individuals should be able to approve requests to buy their data and receive returned value (e.g., money, discount, or free services).

### 4.7. Key Enabling Technologies for PDS Platforms

**Blockchain** can be viewed as a decentralised Internet infrastructure that provides a shared, immutable, and transparent history of transactions. In a blockchain network, a set of miners work together to verify and record transactions and maintain a public ledger [70]. From a technological point of view, integrating blockchains with the development of PDS platforms can provide multiple features. First, blockchains as a decentralised system can provide a robust storage system since there is no central point of failure. In addition, PDS platforms need to provide a unique identity (Self-Sovereign Identity) to associate individuals’ personal data, which could lead to several other benefits, decentralised access control, decentralised data search, and decentralised data marketplace [71]. Moreover, blockchain technology helps PDS platforms with requests related to data authentications, verification, and authorisation.

**Smart contract** has been introduced earlier than Blockchain, but it has been recently associated with Blockchain. This is because smart contracts are a form of self-governance and self-managed transactions that can be executed and stored automatically in the Blockchain, enabling self-governance over data. In the context of PDS platforms, smart contracts can be used as a solution for personal data determination, which refers to the ability to determine the ownership of personal data and the right to use and transfer it [70]. In SOLiD, smart contracts have been transparently defined and enforced data access policy in which individuals and service providers can deploy policies as smart contracts [9].

**Semantic** technologies are used to ease data interoperability, which is regarded as an essential feature of a fully functioning PDS ecosystem. This is because, in reality, PDS platforms need to effectively interact and communicate with various types of data forms, data exchange protocols, systems, heterogeneous devices, etc. Therefore, semantic technologies can facilitate interoperability through semantic annotation, managing access, resource discovery, and knowledge extraction [72]. With semantics technologies, individuals could also transfer and exchange personal data with various entities (e.g., between PDSs). For instance, RML.io (RDF Mapping Language) has been used in a proposed solution that allows individuals to transfer personal data into an interoperable format to their personal data store [73]. Furthermore, semantic technologies are used to link and organise data in decentralised stores based on authorisation methods for granting access to data. In order to automate these processes, Ref. [74], for example, used semantic web-based policy languages which allow expressing rich rules for consent and data requests.

Various other technologies have also been used to enable the existence of PDS platforms, such as Machine Learning and Artificial Intelligence (AI). In this context, the use of machine learning tools have been used to learn how to answer future third-party data requests [75], privacy preference suggestions and personalised privacy settings, and privacy preference enforcement [11]. Users of PDS can also benefit from personalised AI services by providing controlled access to their data or by asking providers to send their AI services into users’ PDS [76].

### 4.8. Existing PDS Platforms

**Figure 3:**
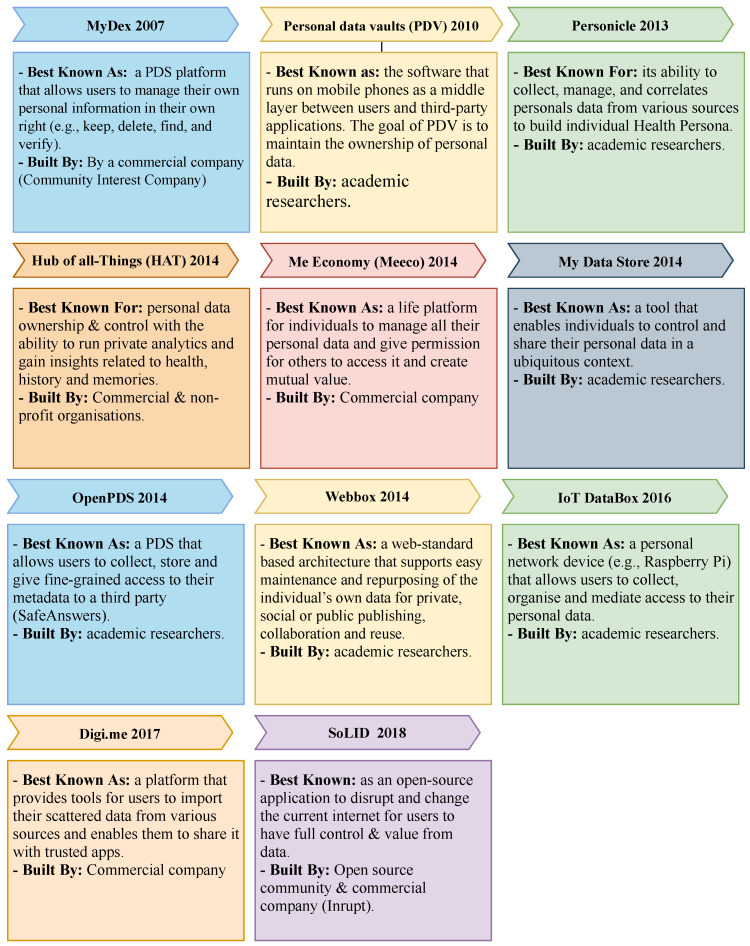
The evolution of the personal data store.

Many PDS platforms have developed over the last two decades. While some of these platforms were built by commercial companies and the open-source community, others were developed as research projects. Each of these platforms has focused on specific features to help grow and adopt the user-centric model. In the following, we will discuss the development of these platforms as depicted in Figure 3.

**Hub of All Things (HAT)** is a decentralised micro-server that gives individuals the full legal right to their data. This micro-server is hosted in the cloud, and personal data can be accessed using various devices [77]. Collected data from various sources can be stored and visualised. In addition, users can install tools (apps) in their micro-server to conduct private analytics and gain insights about their health, e-history, and others. With relevance to data access, users can use some technical tools to transfer their data with their permission and permit app developers to analyse their data. In return, the user can have tangible benefits such as free service. The HAT PDS can only be accessed by the owner (user) and not by HAT because users are considered here as the only controller and processors of the data within the HAT PDS.

**Mydex** is a PDS platform that is designed to enable users to realise the value of their data [78,79]. Users can achieve this goal by allowing app developers or data consumers to access their data. Each time they access a user’s data, they have to pay a transaction fee to the PDS users, and the platform collects a percentage of each data transaction. Mydex is a cloud-based platform on which various apps can be installed. Because of encryption, only users can view data in the PDS account. However, app developers and data consumers can also view specific data once they have the required consent. In addition, the platform provides different data capture mechanisms, and users can fill in their data or let other organisations populate their PDSs.

**Personal data vaults (PDV)** is privacy architecture presented by Refs. [80,81,82]. PDV is software that runs on a mobile phone and communicates with PDV, which works as a middle layer between a user’s mobile phone and the third-party application. PDV works like an online personal data storage, where an individual can upload personal data. It provides storage, authentication, access control mechanisms, and a user interface. The goal of this PDV is to maintain the ownership of the individual’s data. PDV acts as a middle software that allows individuals to control and filter data before being shared with internet service providers. Individuals can also decide what and with whom data will be shared. However, PDV is designed for the mobile phone environment. As a result, stored data are only related to locations, movements, images, texts, and health data.

**Personicle** was presented as a framework that collects, manages, and correlates personal health data from heterogeneous sources and detectors events happening at a personal level [83]. Data is gained from different sensors such as Microsoft Kinect, onboard sensors on mobile phones, and wearable tracking sensors.

**Meeco** is similar to previous PDS in terms of empowering individuals to own and benefit directly from their data [84]. However, Meeco is more focused on helping individuals to gain insights and have the data to negotiable better outcomes.

**MyData Store** is a tool that enables individuals to control and share their data [85]. According to this study, MyData Store is a secured digital space owned and controlled by the user and acts as a repository for personal information. They designed this model to collect, share, and delete personal data on mobile phones. The framework provides a user-centric and data management tool that can be used through the whole lifecycle of individuals’ data, from data collection and use to data trading or monetisation [28].

**OpenPDS** is another framework introduced by Ref. [26] intending to enable individuals to manage their data safely and privately by giving only short answers to third parties and prevent any direct access to the data. This framework is a practical way to protect the privacy of individuals. This framework proved to be viable because it was applied as a novel approach for recommender systems to overcome the limitations of the existing systems [86].

**Webbox** was initially introduced as a web-standard-based architecture that supports easy maintenance and re-purposing of the individual’s data for private, social, or public publishing, collaboration, and reuse [87]. It was also proposed as an alternative solution to the existing online Personal Information Management (PIM) service, which does not enables users to fully control their information in terms of how it can be accessed, stored, and guaranteed (e.g., long-term persistence and security).

**Databox** is an alternative user-centric approach proposed to enable individuals to coordinate the collection processes and the management of their data [38]. Databox allows users to selectively and transiently share personal data with a third party for specific purposes. Later, the IoT Databox model is presented to enable internal and external accountability [42]. The IoT Databox was mainly designed as a physical device for the IoT environment. Data transfer is enabled here, and users can install apps locally. Unlike PDS HAT, Databox assigns the role of the data controller to external parties, such as app developers, when data is transferred out of the Databox, and they would not be transferred when the data is at rest in the device.

**SOLiD** proposed to provide a set of tools for building decentralised Web applications, including the ability for individuals to store and trade their data [88]. In addition, they offer actual data ownership, where individuals can choose where their data is stored and who can access it. Organisations can also benefit from existing data that users have already stored and use such data without needing to build up customer networks.

**Digi.me** provides tools for individuals to import their scattered data from apps and websites. Once data is imported, individuals would take control of the data [89]. They would also be able to search and browse that data and let third-party apps and websites integrate and access it. Digi.me claims that its business model complies with GDPR consent requirements for data processing.

**KRAKEN Project** is a European project that aims to develop a trusted and secure personal data platform. It enables individuals to share trade-sensitive personal data (e.g., educational and health records and well-being data from wearable devices) and their ability to maintain full control and ownership of their data throughout the entire data lifecycle [90]. The project also aims to provide individuals with advanced technological methods such as privacy-aware analytics, self-sovereign identity, and data portability control. KRAKEN, as a personal data platform solution, initially aimed to focus on the health and education sectors.

**PimCity Project** enables individuals to regain control of their personal data by building a platform where individuals can share and trade personal data with businesses and organisations [91]. The project delivers Personal Information Management Systems (PIMS) based on a user-centric model. The project also aims to increase transparency in the online data market by implementing a PIMS development kit (PDK) (e.g., personal data safe and personal consent management) that allows developers to engineer and experiment with new solutions.

**TRUSTS Project** aims to create a secure and trustworthy European market for personal and industrial data [92]. The project was initiated in 2020 by European Union’s Horizon research and innovation research and is based on the experiences of two large national data-sharing projects. The platform aims to connect stakeholders, provide generic functionality, and act as a platform federation between data markets. Furthermore, the platform provides an operational and GDPR-compliant European data marketplace and follows the reference architecture designed by the International Data Spaces (IDS). The platform aims to improve the integration and adoption of future platforms by providing services to identify and overcome legal, ethical, and technical challenges across-border data markets.

## 5. Analysis of Existing Personal Data Stores

PDS platforms provide an alternative way for individuals to regain control over their data. Currently, personal data are collected and processed by big institutions (companies and governments) and app developers. One crucial flaw with this approach is that users usually have very limited visibility over their data in terms of various aspects, including the collection, analysis, and sharing of data. In contrast, PDS platforms provide various capabilities and the needed infrastructure that allows users to collect, analyse, give permissions for data access, and share their data with those interested in it.

Several PDS platforms are available today for individuals to use and control their personal data. Therefore, in the following, we will explore various available PDSs platforms. We intend to analyse these PDS platforms based on their capabilities that empower individuals to control their data [7]. These functionalities can be seen as follows:

1. Ability for individuals to capture and store personal data from different sources.
2. Ability for individuals to process and conduct computation analysis to gain a better understanding of themselves and provide apps that help them achieve that.
3. Ability for individuals to view, monitor and take immediate actions in real-time with aspects related to the control of their personal data.
4. An individuals’ ability to gain social and economic benefits by controlling the disclosure of their personal data based on their terms and preferences.

Based on these essential functions, several existing PDS platforms, readily available for individuals to use, are analysed in the subsequent sections.

### 5.1. Personal Data Capture and Storage

In the digital world, personal data can be generated in various ways, including and not limited to sensors, online web services, and data entry. However, data can be generated automatically by the software and by browsing websites [93]. PDS platforms are supposed to offer individuals tools to collect personal data from various sources. The collected data will then be stored locally in a physical device or the cloud. In addition, individuals should be able to manually enter and store their personal data. Finally, individuals should also be able to delete some or all of their personal data.

### 5.2. Personal or Self-Data Analytics

Unlike the current approach, where personal data is processed and analysed using third-party servers, PDSs offer individuals the ability to perform analytics locally [94]. Users can process and analyse local data stored in their PDSs by installing and executing apps at their PDSs. Depending on the PDS platforms, apps might need to transfer data from a user PDS to app developers to process the data once they have permission to do so. On the other hand, some apps allow users to perform all data processing and analytics locally, but they need user consent to access their data.

### 5.3. Data Access-Control, Data Sharing, and Data Transfer

One major issue with the current internet paradigm is that users can only benefit from web-based services by giving service providers a set of permissions, including indefinite access to their personal data. Users usually have no choice but to limit or stop these permissions without service cancellation. As an alternative approach, all PDS platforms have very restricted terms regarding data access, data aggregation, or data release. This means that data consumers always need to specify why and what type of data needs to be accessed and transferred, and where and how data analytics results will be used. For example, users could limit the number of times their data will be accessed for more security. The primary goal of these restrictions is to give users full control over their data processing and analysis. Similarly, app developers or any interested party in the result of data analytics will not have access to use the raw data since they are not responsible for data management or processing.

In Databox, users can control data access according to their privacy needs and preferences. Users can be more specific in terms of the restrictions of the duration of data source accessibility, how frequently data can be accessed, how data can be read, and other abilities to reduce data dimensionality.

Furthermore, PDVLoc was developed as a model for access control mechanism [80,81]. This framework is designed to share data selectively through a Personal Data Vault (PDV). This framework aims to provide users with flexible and fine-grained access control over their location data. In Ref. [95], another novel architecture system allows an individual to selectively assign access rights to various data consumers by using an authorisation manager. This architecture allows individuals to define data sharing policy using a specific web-based interface. It can also be described as a data-sharing protocol that interacts with all mentioned entities.

The ability to share personal data has many issues such as the right of ownership, storing, and protection. Several solutions in the literature have been presented. For instance, a decentralised identity manager was proposed and tested as a viable solution to these issues [4]. This research provides a PhD project that focuses on the analysis of mental health user requirements, concerns, and expectations for sharing personal data with health providers and others [41]. The findings of this research show that there are some recommendations that designers and app developers need to consider. For instance, the interviewee expresses concerns about the journey of their date if they allow access to it. They also need full control to decide when whom and what level of data can be shared, and they need to have a trusted technological solution (with no data leak) to share the data.

As we mentioned earlier, PDV is a proposed architecture that allows users to define data for sharing and make decisions about with whom data can be shared and at what level of data [81]. Some of the previous research studies only work regarding location. Besides, most of this research works directly with social sites. However, this paper is more concerned about sharing personal data by using personal data stores. For personal data sharing, Ref. [70] propose a personal data determination method based on smart contract and blockchain. This method enables individuals or data subjects to claim the ownership of their personal data and who can access or use it, and how to transfer the data ownership to others. For data sharing using PDS, Ref. [44] proposes a framework to guarantee the authenticity of the shared data in real time and provide transactional privacy in a blockchain network. They argue that in the PDS-System, the shared data is not accessed directly by data consumers who often rely on offline authorisation mechanisms. Their framework solves this problem by allowing data consumers to verify the shared document’s authenticity easily. A similar blockchain mechanism was proposed for OpenPDS [96]. However, they differ in terms of whether personal data is stored in blockchain (OpenPDS) or in PDS.

### 5.4. Monitoring, Visualisation, and Data Trading

Many PDS platforms provide various means for users, including logs, audits, and visualisations, to monitor and have insights about personal data at PDSs and the behaviours of installed apps. This means that users can review and inspect data processing and operation at their PDSs and change their preferences and constraints whenever necessary.

Bell [48] has proposed an artefact (software service) in a fictional data trading scenario. He used agent-based modelling to learn more about individuals’ trading and marketplace behaviours. He presented a personal data trading model for a single person and data trading business. Fictional constructs or objects that emerge from this model have also been discussed. Other researchers address the design of sensing as a service ecosystem where data owners can trade their personal data using the Data Bucket App [19]. HAT also provides individuals with a micro-server that stores data client-side. The primary purpose of HAT PDS is to create a new marketplace for users to trade and gain value over their personal data.

## 6. Architectures for PDS Platforms and Their Components

**Figure 4:**
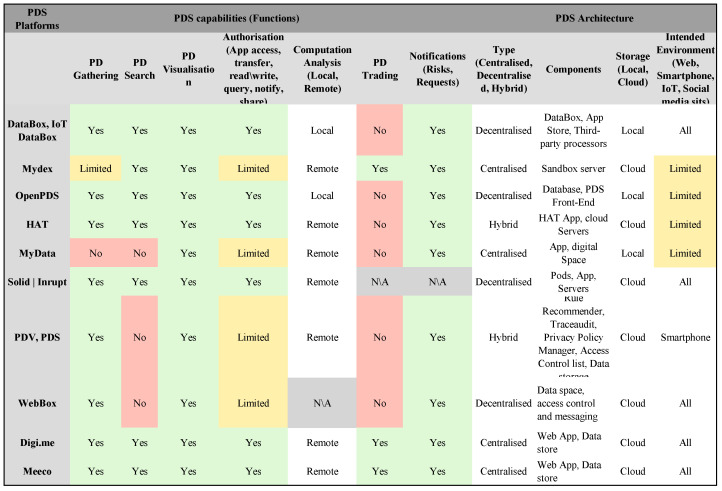
Analysis of the capabilities and architecture in PDS. (HAT [77], Mydex [78], PDV [81], Personicle [83], Meeco [84], MyData [28], OpenPDS [26], Webbox [87], Databox [38], SOLiD [88], Digi.me [89]).

The architecture of PDS platforms can be categorised into three categories: centralised, decentralised, and hybrid [4]. First, we define centralised PDS platforms as when only a central authority manages the service and trust between users and services and mediates trust and legal issues. In contrast, decentralised PDS platforms are characterised by the absence of central authority, but specific methods are used to regulate trust and data exchange. Finally, in hybrid PDS platforms, users and a few reliable authorities shared the role of management and trust (see Figure 4). In addition, PDS platforms are designed to be cloud-based storage or local-based storage. With cloud-based PDS platforms, APIs will act as an intermediate layer and an access point to web-based technologies for third-party developers with a proprietary system. In contrast, local-based PDS platforms require individuals to have a physical device to store their personal data in encrypted form and access it through APIs.

**Figure 5:**
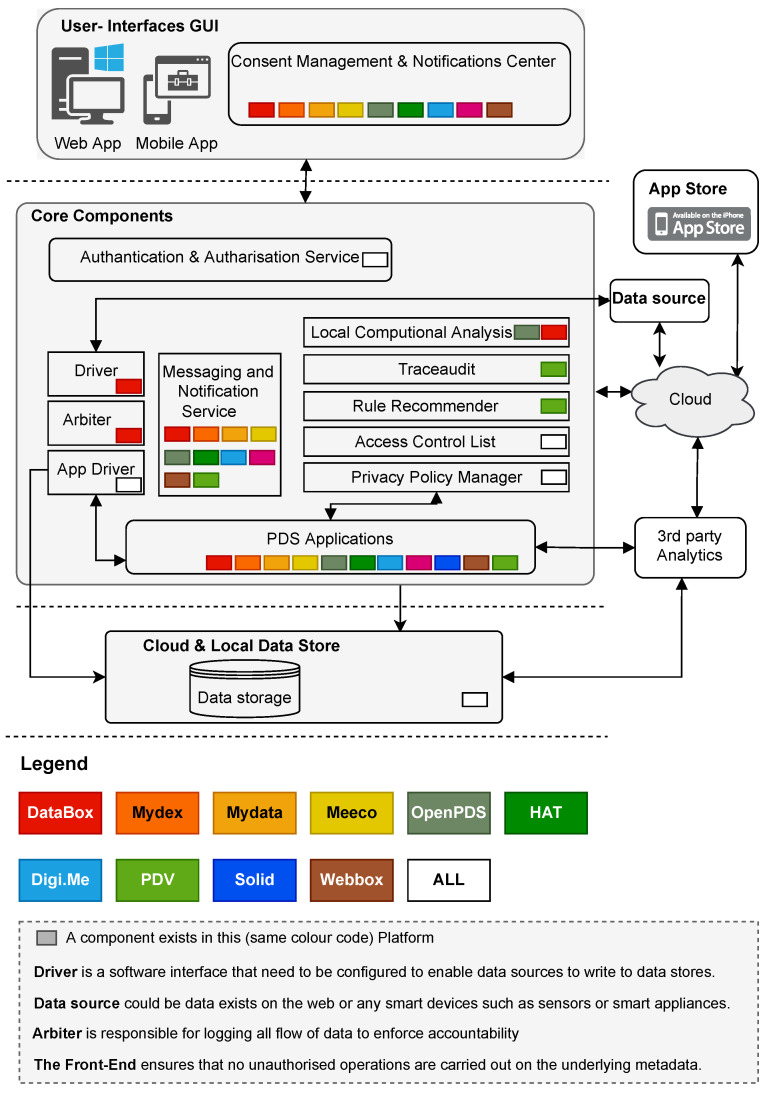
Architectural components of the existing PDS platforms. (HAT [77], Mydex [78], PDV [81], Personicle [83], Meeco [84], MyData [28], OpenPDS [26], Webbox [87], Databox [38], SOLiD [88], Digi.me [89]).

Based on the main aims of these PDS platforms, each PDS platform has logically distinct components. These components are essentially responsible for core functions related to storing data, managing data and access control, managing identity, managing privacy preferences (authentications, authorisations), and providing web interfaces for individuals to manage consents and notifications as illustrated at a high level in (Figure 5). Each PDS platform uses various components, which we will discuss separately in the following sections. The aim is to provide an architectural overview of each PDS platform without going too deep to explain all the technical details.

OpenPDS has a unique architecture to increase an individual’s privacy by answering questions instead of releasing or sharing copies of raw data or anonymised metadata [26]. The framework of this architecture is called SafeAnswer and comprises two separate layers. The first layer includes the database, where storing and processing sensitive data takes place. The second layer (PDS Front-End) uses a privacy-preserving group computation method to anonymously aggregate data related to various users without sharing sensitive data. This architecture is believed to provide one of the safest privacy mechanisms because requests for personal data are always processed and validated by the PDS Front-End and sent back as answers without needing to share the raw data.

Databox has several components [40] including container manager, driver, store, apps, and arbiter. External data sources access the Databox via drivers, and data will become available to apps for processing. Individuals can load Apps from a remote store provided by third parties. Databox is a platform where data from various resources can be accessed and processed locally. The container manager allows access to selected stores by external data processors. It also contains a set of management functions to manage container instances, record all installed drivers and applications (directory), provide interconnection between running components (bridge), and manage the interaction between components and external processors (arbiter).

The MyData architecture aims to provide a standard that enables individuals to easily grant and withdraw consent for data processing [28]. It also aims to enable service creation and provide tools for individuals to track and monitor how their data is being used. Within MyData architecture, there are four core concepts, including the individual as the Account Owner, MyData Operators, Sources, and Sinks. The MyData operator is responsible for hosting MyData accounts which enable digital consent management (authorisation as a service). In addition, MyData Account encompasses the individual’s digital identity, linked services, and authorisations. The source is another important entity that provides Account Owners’ data (only with given authorisation) to one or more Sinks. Finally, Sink is an entity that fetches data (only with authorisation) from one or more Sources and uses the data to produce the agreed services.

As mentioned earlier, the architecture of PDV is designed as a privacy approach that aims to secure sensitive data stored on mobile phones, such as locations, images, and health data. According to PDV architecture, an individual’s data can be stored in secured containers to which only the individual has complete access and control. Based on PDV architecture, there are three mechanisms for managing data policies: granular Access Control Lists (ACLs), a rule recommender, and a traceaudit. Granular ACLs enable individuals to control and selectively share fine-grained location data. The rule recommender provides informed knowledge of the consequences of location data sharing and facilitates the application of privacy policies. Finally, the traceaudit aims to provide frequent reports regarding data sharing and alerts when potential risks are detected.

In Solid architecture, the main component is a pod which refers to a Web-accessible personal online data store where the data of individuals is stored. In this architecture, an individual’s data is managed independently from the applications that generate and consume this data. Some of the existing W3C standards and protocols enable features such as authentication, interactions between application pods, and communications between pods. Solid also uses vital technologies such as decentralised authentication, a global ID space, and global single sign-on. Based on this architecture, applications can gain access to the user’s pod through the identity profile, which is stored on a pod server. It will then follow links initiated by the profile to discover and access individual data on one or multiple pods.

The WebBox architecture assumes that every individual has their own WebBox and HTTP server, which hosts and securely maintains their data and mediates interactions between other WebBoxes. Mainly, there are three components for the WebBox, namely data space, access control and messaging. First, data space is used as a repository for small structured information (data objects). The second main component is access control which is used to authorise and configure data access for users and applications based on predefined sharing policies. Finally, the messaging entity is responsible for notifying and receiving notifications from remote WebBoxes regarding data changes or updates.

### GAIA-X and IDS as Global Architectures for Data Space Ecosystem

The GAIA-X and International Data Spaces (IDS) Reference Architecture are closely aligned with a shared goal to create the next generation of data sharing platforms (Data spaces). Based on this architecture, data spaces can be defined as a broad term that includes any ecosystem of data models, datasets, ontologies, data sharing contracts, and specialised management services (i.e., data stores, centres, repositories) for European companies and their citizens [97]. The aim is also to build a data infrastructure with focus on data sovereignty and creating a trusted data ecosystem where personal data and industrial data can be securely and safely shared among participants (e.g., data owner and data consumers).

However, the GAIA-X project specifically aims to provide a regulatory and technical framework for data infrastructure and service providers [98]. The GAIA-X architecture can be structured into data and infrastructure ecosystems. The former enables data spaces where participants exchange data and smart services such as AI, and big data and analytics are provided. The latter focuses on providing and consuming infrastructure services (e.g., hardware noes, application containers). The architecture also includes components of how data is stored, transferred, and processed. It also defines the participants involved in this ecosystem, such as cloud service providers, network providers, and edge cloud providers.

On the other hand, IDS Reference Architecture Model provides (RAM) a framework to describe the roles that a participant (e.g., individuals and companies) can play in data spaces. The RAM provides a technical description for a data space software architecture. The architecture aims to maintain data security and protection for all involved participants. From a functional point of view, the main components of the IDS RAM are IDS Connector, IDS broker, and IDS clearing House. IDS Connector is the most important building block responsible for ensuring that participants maintain sovereignty over the data [97]. IDS connector acts as an interface between the internal systems of the IDS participants and the IDS ecosystem.

Both can be used as a blueprint for data space implementation [99]. However, Gaia-X uses the "International Data Spaces" Reference Architecture to ensure that data usage controls are provided and compliance is assured. Individuals can benefit from both architectures by guaranteeing privacy and receiving fair value or compensation when they share their personal data.

## 7. Discussion

Since several PDS platforms are designed differently to provide a wide range of functionalities, it is important to evaluate their applicability concerning the above-discussed capabilities in the section and how such PDS platforms are being used. Therefore, our analysis is mainly based on an evaluation framework presented in Figure 4.

HAT PDS is an industry-type platform that can be utilised by individuals, developers, and organisations from different countries worldwide. This platform can also be viewed as one of the best well-designed PDS solutions for individuals. As we discussed in Section 4.8, this platform provides a decentralised micro-server for individuals to collect personal data from various resources on the Internet by linking their HAT Personal Data Account (PDA) with web-services (e.g., social media accounts, Fitbit, and Spotify). Furthermore, individuals can view, search, share, and soon analyse personal data to gain better insights. Unlike organisations that need to pay fees, the platforms do not charge individuals when they offer products and services (universal ID, authentication, grants ownership, and control of personal data). Similarly, Meeco and Digi.me platforms provide tools for individuals to access, control, and securely exchange personal data with participants in the data ecosystems. However, these two platforms are not as technologically mature as the HAT PDS platform, which provides better integrated apps and tools for acquiring personal insights.

Similarly, Mydex has already been used by many individuals, service providers, and governments in different counties. With this platform, individuals can store their data in their own PDS and use it for exchange services such as managing chronic health conditions, accessing debt advice, and assuring their identities. In terms of capabilities and applicability, this platform is one of the most mature PDS platforms that empower individuals to control their personal data.

In contrast, OpenPDS is built as a personal metadata management framework that allows individuals to collect, store, and give fine-grained access to their metadata. However, OpenPDS cannot be considered a stand-alone PDS platform that provides an independent data-sharing ecosystem (e.g., Mydex or HAT) that enables individuals to share and trade their personal data. Instead, this platform can be seen as a service (SaaS) for improving the privacy and security of personal metadata. This service can be installed in a personal server or a virtual machine to manage and view data access requests. Similar to OpenPDS but with different system architecture, PDV was proposed as a privacy architecture by which individuals regain ownership of their data. However, PDV was limited to location data in the context of smartphones.

In Databox, although the platform is designed to manage data from various resources, data cannot be stored locally. The platform is decentralised and aims to provide all the needed capabilities except for data trading. As we mentioned, this platform was built as a research project (preliminary prototype) with many unresolved challenges. In the same vein, MyData and WebBox were built by researchers based on a user-centric approach. However, they are very limited in terms of their capabilities and potential application in real-world settings.

## 8. Challenges and Future Directions

Research related to PDS platforms is still in its infancy, but rapid development and promising achievements can be seen. Nevertheless, PDS platforms still face several challenges before reaching a reasonable maturity level. As shown in Table 3, we divided these challenges into three categories, including social, legal, and technical challenges. Each challenge signifies several potential directions for future research. In the following sections, we will discuss each category in more detail.

### 8.1. Social Challenges

One of the social concerns about the PDS model is how to increase the individual’s adoption and use of this model when most ordinary users have different perceptions of privacy and security risks. In addition to those individuals who need to see this model’s value and the troubling make. Individuals are usually only interested in trying new platforms with new and tangible benefits. For example, although no PDS platform currently requires individuals to pay fees for using their platform, there are still some hesitations about joining due to the lack of trust and other issues. Furthermore, recent research shows individuals’ lack of interest in using and adopting PDS platforms, which may demotivate PDS providers to build new or improve the current functionalities of PDS. These challenges can be mediated by offering transparent, flexible and secure PDS platforms with tangible and distinct benefits for individuals [24].

### 8.2. Legal and Regulatory Challenges

In response to the legal requirements for the modern data economy, governments and legislative bodies around the world have started introducing regulations (e.g., GDPR) to protect our data. These regulations could be a driving force for individuals to trust and join PDS platforms. However, there are several legal challenges that PDS platforms need to overcome. The first challenge revolves around how to identify data controllers and processors [4]. In other words, PDS platforms need to determine the purpose for which and how personal data is processed. This is especially important for individuals interested in sharing their data with third-party developers or apps.

Another issue is related to the fundamental rights that individuals need to exercise over their data. For example, in Art 16 and 17 of the GDPR, data subjects have the right to rectify, be forgotten, and withdraw their consent at any time. Although some PDS platforms might allow users to exercise some of these rights, there are situations where it could be difficult or impossible to achieve that, especially in a decentralised environment. Last but not least, GDPR enforces data processors (e.g., app developers) platforms to be transparent. This includes purpose specification, recipient, transfers, and salient details of automated processing. Thus, PDS platforms need to provide mechanisms to show the potential risks related to data access, processing, and sharing. In some existing PDS platforms, some limited transparency tools are designed to articulate risks related to apps, and dashboard notifications, which allow users to review the status of data processing, data processing operations, and the history of apps operations.

### 8.3. Technical Challenges

PDS platforms are determined to give individuals a set of technical capabilities that enable them to regain control over their data for a long time. However, this objective imposes some technical challenges that need to be tackled. These challenges can be divided into two categories regarding the architectural design of PDS platforms and personal data management.

A major technical challenge associated with the design of PDS platforms is to build a technical solution with a high level of interoperability. This means that the PDS architecture must cooperate seemingly with other devices, systems, and technologies without difficulty or restriction. This also includes the interoperability of data between different PDS providers. To lessen this issue, organisations must work with different organisations in any sector and make agreements about various things such as standards, protocols, and others.

In addition, PDS platforms need to provide methods for individuals to capture and understand their privacy preferences in different contexts [24]. Similarly, continuous adaptations for users’ privacy preferences that may change over time must be technically addressed. Regarding data trading, PDS platforms should be able to filter, test, and recommend the most appealing offers for the data owners based on their privacy preferences, and expectations. However, the key challenge here is the ability to provide means for data owners to engage and negotiate offered rewards by data consumers and potential risks associated with disclosing personal data. These privacy risks need to be carefully analysed and presented to data owners in a simple and meaningful way (e.g., better smart UIs). In addition, those individuals with little technical or no experience should be able to handle the complexity of managing data security and longitudinal maintenance with ease [14].

Another technical challenge is related to where personal data is processed. Currently, well-developed PDS platforms provide only cloud-based architecture. However, it would be even better for individuals to have another option to store and process their personal data locally (personal server or machine). This means that they do not need to transfer their raw data to a third party to perform data analytics. Further, individuals will improve their privacy and reduce potential security risks. Other benefits of local control are potential computational advantages, decreasing latency, enhancing resilience, decreasing network traffic and availability, and access to data. Consequently, PDS platforms should enable users to exercise their rights to limit and minimise data distribution, aggregating data on the box and only returning the results of processing to data consumers. HAT expose raw data to applications and fail to limit the potential risk of personal data misuse or the potential use of data for unintended and not planned purposes [42]. Even though this is a significant issue that needs to be considered, some PDS platforms expose raw data and allow third-party organisations and apps to access and transfer personal data.

Finally, PDS platforms need to provide technical solutions for individuals who have the right to own and control specific data (shared ownership). This is very obvious in the environment of IoT where several people (e.g., family members) own one device, sensor, or home appliance. All of them are expected to collect data related to all of them. As a result, all these people must express and determine their data access and privacy preferences. Thus, PDS platforms need to address this challenge by developing tools to manage data access when shared data ownership exists.

## 9. Lessons Learned

**More added-value is needed:** PDS platforms are focused on providing tools for individuals to enjoy the benefits of managing and controlling their personal data. This includes the ability for individuals to conduct self-analytics and self-reporting. Besides, individuals are promised to have the ability to share and manage access to their data. Notably, these benefits might be enough for some individuals. However, to increase the level of adoption, PDS platforms should also be able to provide tangible value and a better experience. One possible way to do this is by creating a transparent market where individuals can negotiate the direct or indirect value of giving access to organisations or app developers. They also need to be able to assess the value of their data independently. The direct value is a small amount of money, discounts, or free products. On the other hand, the indirect value is the ability of organisations to deliver more relevant, personalised and customised services or products. Currently, only basic tools for sharing data and managing data access are provided as simple on and off buttons. Consequently, these tools need to specify the level of raw data being shared and potential risks.

Providing solutions for major problems we face today could also be perceived as added value by individuals. One example of this is related to personal data breaches. Personal data are no longer safe and secure because many prestigious companies such as Facebook and LinkedIn could not prevent hackers from exposing data related to millions of people. Meanwhile, PDS platforms could be a better solution to keep our data safe and with fewer security risks.

**Capabilities and architectural components:** We observed that PDS platforms have different frameworks. For instance, some PDS platforms are focused on personal data stores where individuals essentially could have actual ownership over their data (e.g., HAT). This means that individuals can choose where data is stored and who can access it. On the other hand, some PDS platforms have decided to concentrate on the personal data market with a vision that allows users to gain value from their personal data by sharing or selling it to businesses, governments, and social network sites (e.g., Meeco). Nevertheless, all PDS platforms share a central purpose that evolves around building data stores that allow individuals to collect, store, and give access to other organisations.

We also learned that most PDS platforms share more similarities than differences in architectural components and functions. As a result, multiple platforms might offer substantial functionality and have unique architectural components. For instance, it would be tempting to have PDS platforms that are locally controlled but still have all the cloud-based functions. This means individuals will have better control over data travelling from their devices to the cloud. At the same time, this might create severe issues for those individuals with no technical experience, but with a high level of automation, this problem might be lessened.

## 10. Implications

Our review of the body of literature and existing PDS platforms provides several implications for researchers and anyone interested to know about the current state of PDS platforms. From a research perspective, we did a comprehensive review of research related to personal data stores in terms of their capabilities and functions. As such, we discussed research studies related to PDS platforms and how they have evolved over the last two decades from simple personal document storage to very sophisticated platforms that allow individuals to control their personal data. This review also provides a complete analysis of existing PDS platforms, which could be very useful for researchers to have an overview of their aims, architectures, and capabilities. This contributes to the literature by better understanding the similarities and differences between PDS platforms and their applications. This review revealed a need for further research around multiple research areas, such as adopting and accepting PDS platforms, as well as many research opportunities related to the technical challenges of PDS platforms. Moreover, this review recognised the importance of data value exchange in developing PDS platforms. Besides controlling personal data, individuals need to be able to share their data with data consumers and receive direct or indirect returned value. Future research should investigate the legal aspect of data trading in PDS platforms. Finally, from a practical perspective, this review uncovers the need for evaluating existing PDS platforms in terms of their system performance, ease of use, reliability, and security.

## 11. Limitations

There are several limitations of this review. First of all, although we follow a comprehensive search methodology, this review is limited by a selection of databases and search queries, which may not be sufficient to retrieve all the possible references related to PDS platforms. As such, we do not claim to have covered and identified all related references, although we believe that our results give a detailed and inclusive view of the current literature. Further, the categorisation of topics related to PDS platforms was based on a manual analysis approach. Thus, some degree of subjectivity is inevitably anticipated. Another limitation of this review is that all PDS platforms mentioned here were analysed based on original references. We did not test their capabilities or performance in real-life settings.

## 12. Conclusions

The potential and expectations of PDS platforms have incredible benefits. We expect these benefits to be valuable to individuals, organisations, and societies. While PDS platforms focus on supporting individuals to regain control over their data, organisations would be pleased to have access to clean, rich, and safe data. This clean data would allow organisations to be more cost-effective and have an efficient business process. However, PDS platforms still need to deal with many challenges and issues before they can be successfully and widely adopted. Therefore, this survey aims to explore this area by focusing on recently published research articles. In particular, this report intends to find out what research has been conducted in the area and the main issues and challenges facing the development and adoption of PDS.

Towards this aim, this survey has also explored various research aspects of PDS, including value, architecture and the capabilities of PDS platforms. Next, based on PDS architectures, we summarised their core functionalities. In terms of challenges, we discuss three types of challenges. The first is social challenges, mainly about the user’s perception of the adoption of PDS platforms. Another major challenge relates to the ability of PDS platforms to meet legal requirements and recommendations such as GDPR regulations. Last but not least, PDS platforms can be viewed as an emerging technology that needs to be technically improved. This means PDS designers and developers need to solve a set of technical issues regarding data flow management between systems and applications, automatic and semi-automatic validation of processes performed by PDS platforms, data access and portability, and the ability to deal with the changing effects on personal data over time. We aim to address some of these issues and challenges in our future work. We can use this survey to summarise research aspects related to PDS and address the challenges for researchers and participants in this area.

## Figures and Tables

**Table 1:** Terms used for online search.

Category	Terms
General	Personal data store (PDS) PDS OR Personal Informatics (PI) PDS OR personal data management (PDM)
Specific	PDS OR PDM OR PI AND (design) PDS OR PDM OR PI AND (architecture) PDS OR PDM OR PI AND (functions) PDS OR PDM OR PI AND (data sharing) PDS OR PDM OR PI AND (functions or capabilities) PDS OR PDM OR PI AND (functions)

**Table 2:** Taxonomy of topics related to Personal Data Stores PDS.

Theoretical Aspects	Benefits	[21,24,25]
	Models	[5,18,26,27,28]
	PI	[29,30,31,32]
	PDM	[33,34,35,36]
	Regulations	[24,37]
	Challenges	[19,25,38]
Technical Aspects	Data sharing	[39,40,41,42]
	Access-control	[43,44]
	Data privacy	[45,46,47]
	Data storage	[24]

**Table 3:** Categories of the issues and challenges facing the development of PDS.

Social Challenges	- Lack of interest among individuals to use PDS. - No tangible experience to attract users. - Lack of trust in PDS providers. - Lack of technical experience or expertise for managing and securing data.	[13,14,58]
Legal Challenges	- The determination of data controllers and processors. - Compliance with GDPR regulations allowing individuals to exercise their rights. - Understandability and adaptability of user privacy preferences.	[4,13,19,37]
Technical Challenges	- Data interoperability. - User consent management. - Ease and automation for users with no technical knowledge. - The ability to offer creative tools for data visualisation and analytics. - The effects of the continuous change of personal data and technologies. - The process of integrating all personal data that is collected from various sources.	[13,14,19,26,50]

## Data Availability

Not Relevant.
